# Hypothermia Protects and Prolongs the Tolerance Time of Retinal Ganglion Cells against Ischemia

**DOI:** 10.1371/journal.pone.0148616

**Published:** 2016-02-05

**Authors:** Maximilian Schultheiss, Sven Schnichels, Thoralf Hermann, Jose Hurst, Marita Feldkaemper, Blanca Arango-Gonzalez, Marius Ueffing, Karl U. Bartz-Schmidt, Guenther Zeck, Martin S. Spitzer

**Affiliations:** 1 Centre for Ophthalmology, University Eye Hospital Tübingen, Tübingen, Germany; 2 Natural and Medical Sciences Institute at the University Tübingen, Reutlingen, Germany; 3 Institute for Ophthalmic Research, University of Tübingen, Tübingen, Germany; Universidade Federal do ABC, BRAZIL

## Abstract

**Purpose:**

Hypothermia has been shown to be neuroprotective in the therapy of ischemic stroke in the brain. To date no studies exist on the level of the inner retina and it is unclear if hypothermia would prolong the ischemic tolerance time of retinal ganglion cells, which are decisive in many ischemic retinopathies.

**Methods:**

Bovine eyes were enucleated and stored either at 21°C or 37°C for 100 or 340 minutes, respectively. Afterwards the globes were dissected, the retina was prepared and either the spontaneous ganglion cell responses were measured or the retina was incubated as an organotypic culture for additional 24 hours. After incubation the retina was either processed for histology (H&E and DAPI staining) or real-time PCR (Thy-1 expression) was performed.

**Results:**

Hypothermia prolonged ganglion cell survival up to 340 minutes under ischemic conditions. In contrast to eyes kept at 37°C the eyes stored at 21°C still showed spontaneous ganglion cell spiking (56.8% versus 0%), a 5.8 fold higher Thy-1 mRNA expression (not significant, but a trend) and a preserved retinal structure after 340 minutes of ischemia.

**Conclusion:**

Hypothermia protects retinal ganglion cells against ischemia and prolongs their ischemic tolerance time.

## Introduction

Central retinal artery occlusion leads to irreversible vision loss within hours (h)[1]. Up to date, no causative treatment exists for this ophthalmologic urgency. Past trials using intra-arterial and intravenous thrombolysis could not confirm their efficacy[2,3], although previously published retrospective studies showed encouraging results[4,5]. Nevertheless, it has been observed, that thrombolysis finally leads to reperfusion of the retinal circuit, but apparently the timespan in clinical practice until reperfusion of the retinal circuit occurs, seems to be too long. Therefore, like in ischemic stroke, the crucial factor for the functional result after thrombolysis is the timespan of ischemia[6]. By reducing this timespan to a minimum, thrombolysis as therapy could probably also work in central retinal artery occlusion. In order to increase the potential therapeutic window, we are seeking for substances or procedures, which prolong the tolerance time of the retina to an ischemic insult.

Hypothermia, understood as temperature below 37°C, is supposed to be neuroprotective in cerebral stroke and to increase the tissue tolerance to ischemic insults[7]. At the moment a large efficacy trial in ischemic stroke—the European Stroke Research Network for Hypothermia (EuroHYP)-1 trial—is recruiting up to 1500 patients in order to determine if hypothermia can improve the functional outcome (further information at http://www.eurohyp1.eu/). Hypothermia can prolong the tolerance time to an ischemic insult presumably in every tissue. However, no neuroprotective treatments at the retina have been established so far in the clinics. Comparable to cerebral stroke, hypothermia could protect the retinal ganglion cells during a central retinal artery occlusion. To consider hypothermia as possible effective treatment for acute retinal ischemia, its application should preserve the retina long enough allowing that a curative therapy like fibrinolysis could be performed.

Hypothermia as neuroprotective mechanism at the retina has been already evaluated and proved to be protective[8,9,10]. Earlier investigations always focused on a single time point and thus, important clinical questions remain open[8,9,10]. It is therefore unclear if hypothermia opens a realistic therapeutic time window for causative therapies like thrombolysis. Additionally, no studies exist on the level of retinal ganglion cells, which are the cells of the retina most vulnerable to ischemic insults of the inner retina[11,12].

We therefore conducted a study, in which the effects of hypothermia on retinal function and ganglion cell survival under ischemic conditions were investigated. The shortest ischemic period was 100 minutes (min), because according to a study of Hayreh et al. beyond 97 min significant irreversible damage of the inner retina occurs[13]. Furthermore Hayreh et al. showed, that after 240 min of ischemia no functional recovery can be observed after reperfusion anymore[13]. The aim of our study was to investigate, whether hypothermia extends the retinal tolerance time against ischemia. Therefore, we added additional 100 min to the reported 240 min. According to the published data, the retinal ganglion cells should be dead or dying by then. If hypothermia would preserve the retinal function up to that time point, hypothermia would prolong the retinal tolerance time against ischemia and could possibly open a clinically realistic therapeutic window for a causative therapy (e.g. lysis).

The chosen temperature for hypothermia was 21°C, because in the clinic irrigation solutions used for vitrectomy are stored at room temperature. When a high infusion rate is employed during vitrectomy the temperature at the inner retina decreases to nearly 21°C and the retina is not damaged by this temperature. Unlike in systemic hypothermia that has been studied for neuroprotection in stroke patients there is no risk for systemic hypothermic side effects such as cardiac arrhythmia. Therefore we hypothesized that as 21°C is not harmful to the retina this temperature might be more neuroprotective than 30 to 34°C.

## Materials and Methods

### Preparation of retinal biopsies

Bovine eyes were obtained from the local abattoir (Gärtringen; Schlachthof e.G., Germany). At this abattoir, animals are butchered for food production. No additional animals were killed for this study. The permission to use this otherwise discarded tissue was obtained from the local regulatory authorities (Az.34-9181.30 from the Landratsamt Böblingen, Germany issued in 2006). Ten min after the cow`s death, eyes were enucleated by the abattoir’s veterinarian. The globes were stored either at 21°C or 37°C for 100 or 340 min respectively. After arriving at the lab the globes were cleaned of additional tissue down to the sclera. Then the eyes were disinfected in 10% iodine diluted in 0.9% NaCl (Braun, Germany) for 5 min. After disinfection the globes were washed twice for 2 min using 0.9% NaCl. Thereafter the eyes were dissected and retinal biopsies were prepared under sterile conditions.

Dissection was performed after 100 and 340 min of anoxic ischemia. The preparation of the retina was performed in Dulbecco`s modified Eagle`s medium (Invitrogen-Gibco; Rockville, MD), which was not saturated with oxygen.

Retinal biopsies were taken at a distance of 8 mm from the optic nerve. The biopsy locations were superior of the optic nerve for real-time PCR, temporal for histology and inferior for the retinal ganglion cell recordings. By using defined biopsy-locations the amount of RGCs in the analyzed retinal region was constant for each assay. Additionally one bovine eye could serve as source for different assays. Retinal biopsies were placed on an insert with a permeable membrane (Millipore AB, Solna, Sweden; PIHA03050) facing the vitreous side upwards. Under the permeable membrane, R16 serum free culture medium (Invitrogen Life Technologies, Paisley, UK; 07490743A) was used for cultivation. Retinal biopsies for real-time PCR and histology were cultivated at 37°C in an environment containing 5% CO_2_ for 24 h, the retinal biopsies for measuring spontaneous ganglion cell responses were measured directly after preparation.

After cultivation the samples were frozen using liquid nitrogen. For the real-time PCR samples were stored at -80°C. For Histology unfixed samples were embedded in Tissue-Tek^®^ O.C.T.^™^ (Sakura, Nehterlands), frozen and stored at -20°C.

We did not randomize our eyes during the retina preparation and the MEA-recordings. However, neither, the person who performed the Thy-1 mRNA nor the person who performed the MEA-recordings was aware to which treatment group the respective samples belonged.

### Recording of ganglion cell activity in bovine retina

The retinal biopsies for the 10 min control group were processed directly at the abattoir. The biopsies for the other groups were prepared as described above.

The recordings of RGC-activity was performed like previously described[14]. Briefly, retinal tissue was mounted, ganglion cell-side down, on a microelectrode array (MEA) comprising 60 TiN electrodes (Multichannelsystems MCS GmbH, Reutlingen). The electrodes of this MEA are arranged in an 8 x 8 grid with 200 μm spacing between the electrode centres. Spontaneous activity of the ganglion cells was recorded for 10 min after an initial adaptation period of 10 min. During the recording the retina was continuously superfused with carbogenated Ames’ medium (Sigma Aldrich) at 35–37°C. Data were amplified, filtered (0.3–3 kHz) and saved for offline analysis. Ganglion cell activity, the timing of extracellular spike waveform, is detected from raw recordings if they exceed six times the root mean square (*rms)* noise on the corresponding electrode. Correlated timestamps among different electrodes are eliminated to avoid duplicates. Only electrodes with a mean average spike rate of 1 Hz (1 spike/sec) are counted as “active” electrodes (i.e. electrodes picking up ganglion cell spikes). The mean number of active electrodes for each preparation was evaluated. This number is an estimate of the average activity within the investigated retinal portion.

### Real-time PCR

Tissue preparation and cultivation was performed like described above. Only for the 10 min of ischemia control group the eye balls were stored and processed on ice, in order to avoid mRNA degradation on the way to the lab.

From the retinas the mRNA was isolated and cDNA was synthesized and purified using MultiMACS cDNA Synthesis Kit according to the manufactures protocol (MultiMACS mRNA Isolation Kit (8x12), Miltenyi Biotec #130-092-520, Germany)).

Aliquots of cDNA (5 ng) were analyzed in duplicate reactions by qPCR using 1 μM gene-specific primers and Sso Advanced Universal SYBR Green Supermix (BIORAD; California—USA) in a total volume of 20 μl. PCRs were carried out in a CFX96 and analyzed with CFX Manager Software, version 3.0 (BIORAD). Relative expression levels were calculated as previously described[15] with ß-actin as a reference gene. Briefly explained, the relative expression ratio of a target gene is calculated based on the amplification efficiency and threshold cycle in comparison to a reference gene. The following gene-specific primers were (designed with Primer 3 plus software (http://www.bioinformatics.nl/cgi-bin/primer3plus/primer3plus.cgi)) used: Thy1 (F: 5´-ctcggcaccatgaaccct-3´, R: 5´-agacgaaggctctggttc-3´), ß-Actin (F: 5´-ctcttccagccttccttc-3´; R: 5´-gggcagtgatctctttct-3´).

### Histology

Frozen retinas were cut on a cryostat (12 μm sections). Staining with haematoxylin and eosin (H&E) as well as with 4′,6-Diamidin-2-phenylindol (DAPI) was performed to view the retinal structure. For DAPI staining tissue was first fixed with cold methanol. Sections were blocked in 10% BSA and stained with DAPI. H&E staining was performed on unfixed tissue to visualize the retinal structure. Photographs were taken with an Axiovert 135 fluorescent microscope (Zeiss, Göttingen, Germany) which used the AxioVision 4.6 software (Zeiss, Göttingen, Germany).

### Statistics

Statistical analysis was performed using JMP^®^ (SAS Institute Inc., Cary, NC, USA). To test for differences between the groups the Wilcoxon-Kruskal-Wallis test was used for the MEA-data. A two-sided t-test was used for the real-time PCR data. Additionally we performed for the two-sided t-test a Bonferroni adjustment as multiple testing was performed. Differences were considered to be significant at p<0.05 and after Bonferroni adjustment at p<0.025.

## Results

### Spontaneous retinal ganglion cell responses

**Fig 1:**
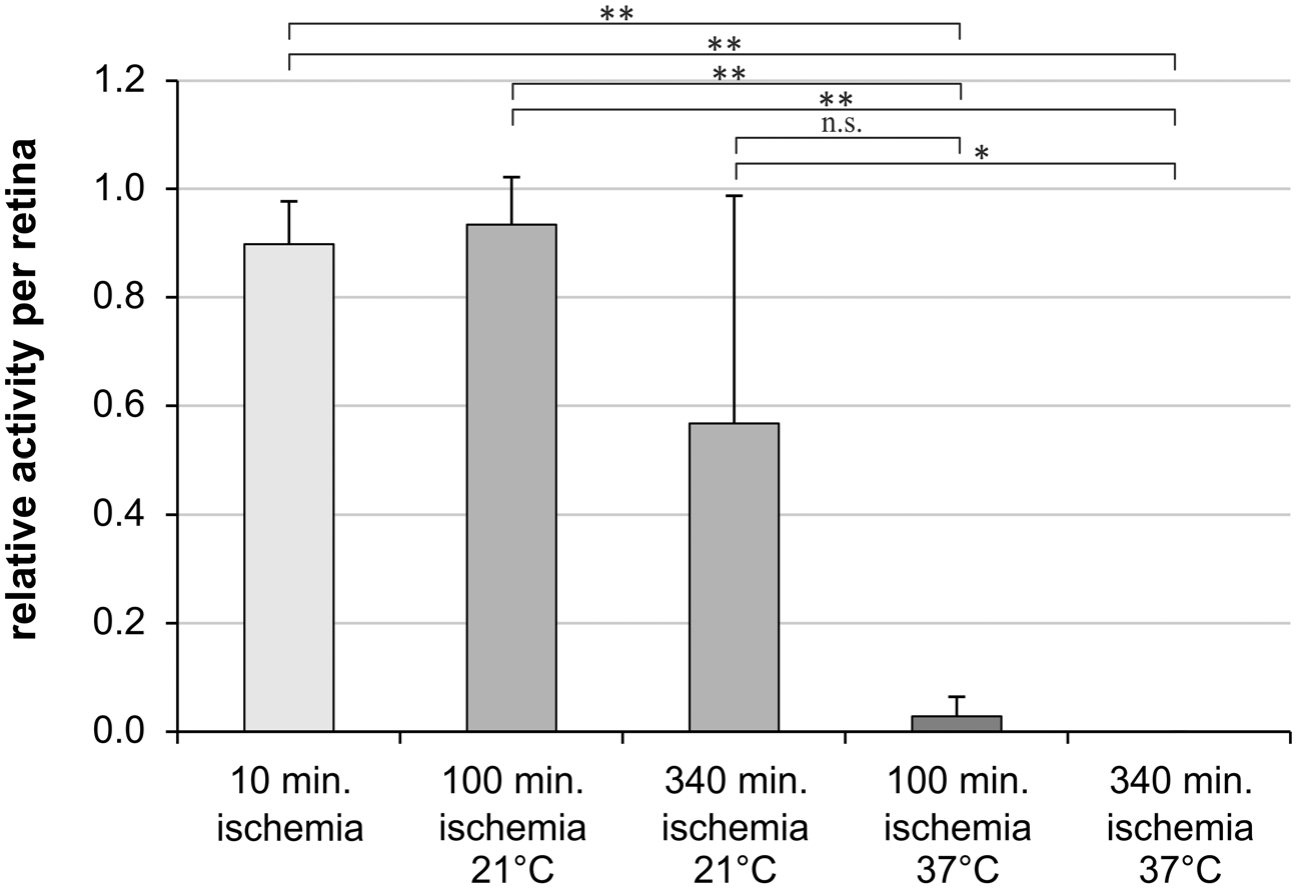
Spontaneous retinal ganglion cell responses are preserved under hypothermic conditions. Bar graph represents the relative amount of electrodes measuring spontaneous retinal ganglion cell activity per retina. Spontaneous action potentials of retinal ganglion cells were measured simultaneously by the 60 electrodes of a Multi-Electrode-Array (MEA). Data are depicted as mean ± SD with * p<0.05 and ** p<0.01; n = 6 retinas.

After 100 min of ischemia the number of spiking retinal ganglion cells was not altered in the eyes stored at 21°C when compared to the freshly prepared eyes which were enucleated within 10 min after death (Fig 1). In contrast after 100 min of ischemia and storage at 37°C the spontaneous retinal ganglion cell responses were statistically significant reduced (Fig 1). Additionally, only in the eyes which were stored at 21°C, but not in the eyes stored at 37°C, spontaneous retinal ganglion cells spiking was observed even after 340 min of ischemia (Fig 1).

In detail, in the retinas with 10 min of ischemia, 89.8% ± 8.9% of the electrodes recorded spikes from retinal ganglion cells. In the retinas from eyes stored at 21°C, 93.4% ± 9.7% (the data had a non-Gaussian distribution) of the electrodes recorded spikes after 100 min of ischemia. The percentage of electrodes recording ganglion cell activity dropped to 56.7% ± 40.9% after 340 min of ischemia (Fig 1). In contrast, in the eyes stored at 37°C only 2.8% ± 4.1% of the electrodes detected retinal ganglion cell activity after 100 min of ischemia. After 340 min of ischemia at 37°C no ganglion cell spikes at all were detected. The attachment of the retina on the micro-electrode array was carefully monitored by analyzing the mean noise level[16], to prevent misinterpretation of the results caused by inhomogeneous interfacing and thus recording of smaller areas.

### Thy-1-Expression

Thy-1 expression levels were normalized to β-actin. The arbitrary units were set as 1 for the samples with 10 min of ischemia and no cultivation for 24 h. These eyes served as control group, as it was not possible to receive the eyes faster than ten min after death.

**Fig 2:**
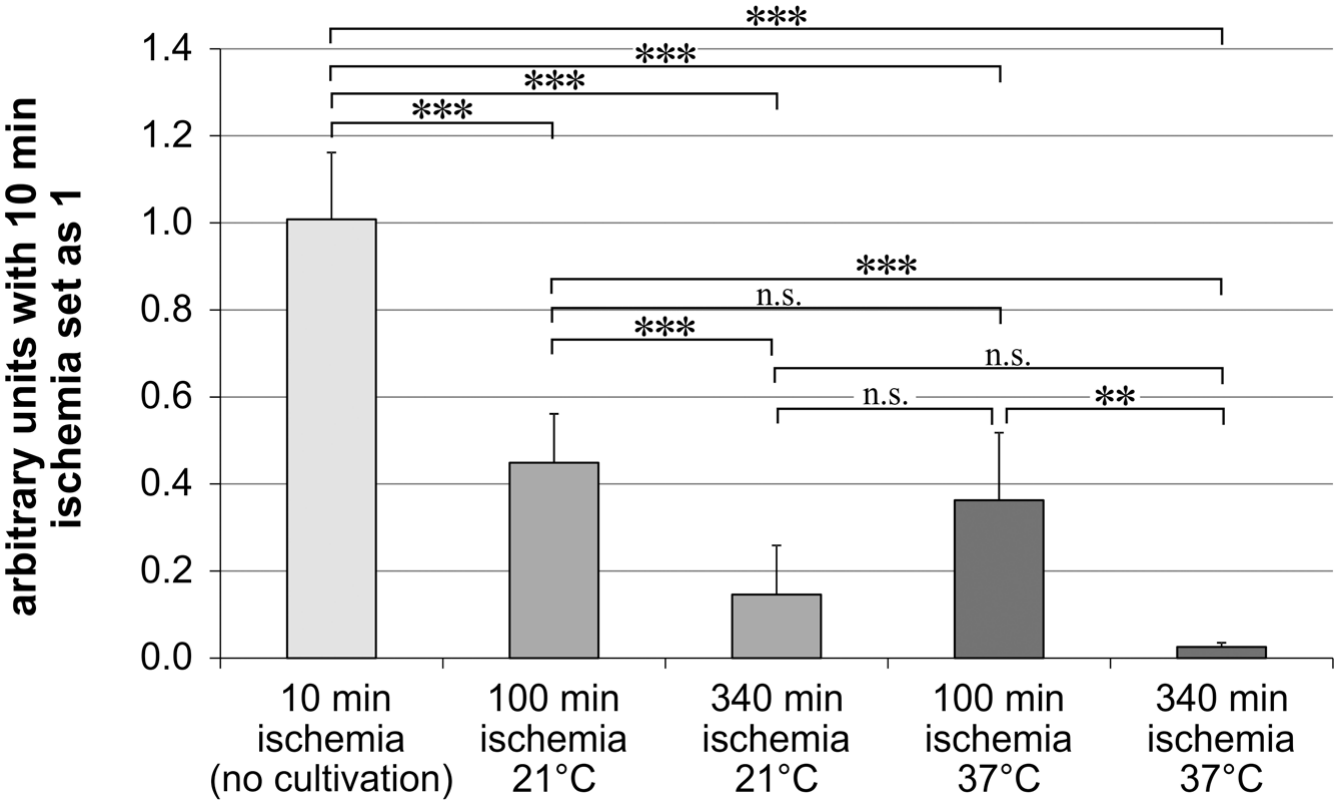
Thy-1 mRNA expression is higher under hypothermic conditions. Bar graph represents the Thy-1 mRNA expression levels in arbitrary units after normalizing them to the samples with 10 min of ischemia. Thy-1 mRNA expression were measured by real-time PCR. Data are depicted as mean ± SD, with ** p<0.01 and *** p<0.001; n = 5–6 retinas.

Longer ischemia times resulted in a lower Thy-1 expression (Fig 2). In our control-group with 10 min of ischemia and no cultivation for 24 h, we observed the highest Thy-1 expression (1.01 ± 0.15). This group was followed by the groups with 100 min of ischemia (100 min ischemia at 21°C: 0.45 ± 0.11; 100 min ischemia at 37°C: 0.31 ± 0.08) and the lowest expression was seen in the groups with 340 min of ischemia (340 min ischemia at 21°C: 0.14 ± 0.11; 340 min ischemia at 37°C: 0.03 ± 0.01).

The eyes stored at 21°C showed after 100 and 340 min of ischemia significantly higher Thy-1 mRNA levels than the eyes stored at 37°C in the two-sided t-test (p<0.05). However after Bonferroni adjustment it was only a trend at both temperatures (p>0.025). Nevertheless after 100 min and 340 min of ischemia the Thy-1 expression was 1.5 respectively 5.8 fold higher when the eyes were stored at 21°C (Fig 2).

### Histology

**Fig 3:**
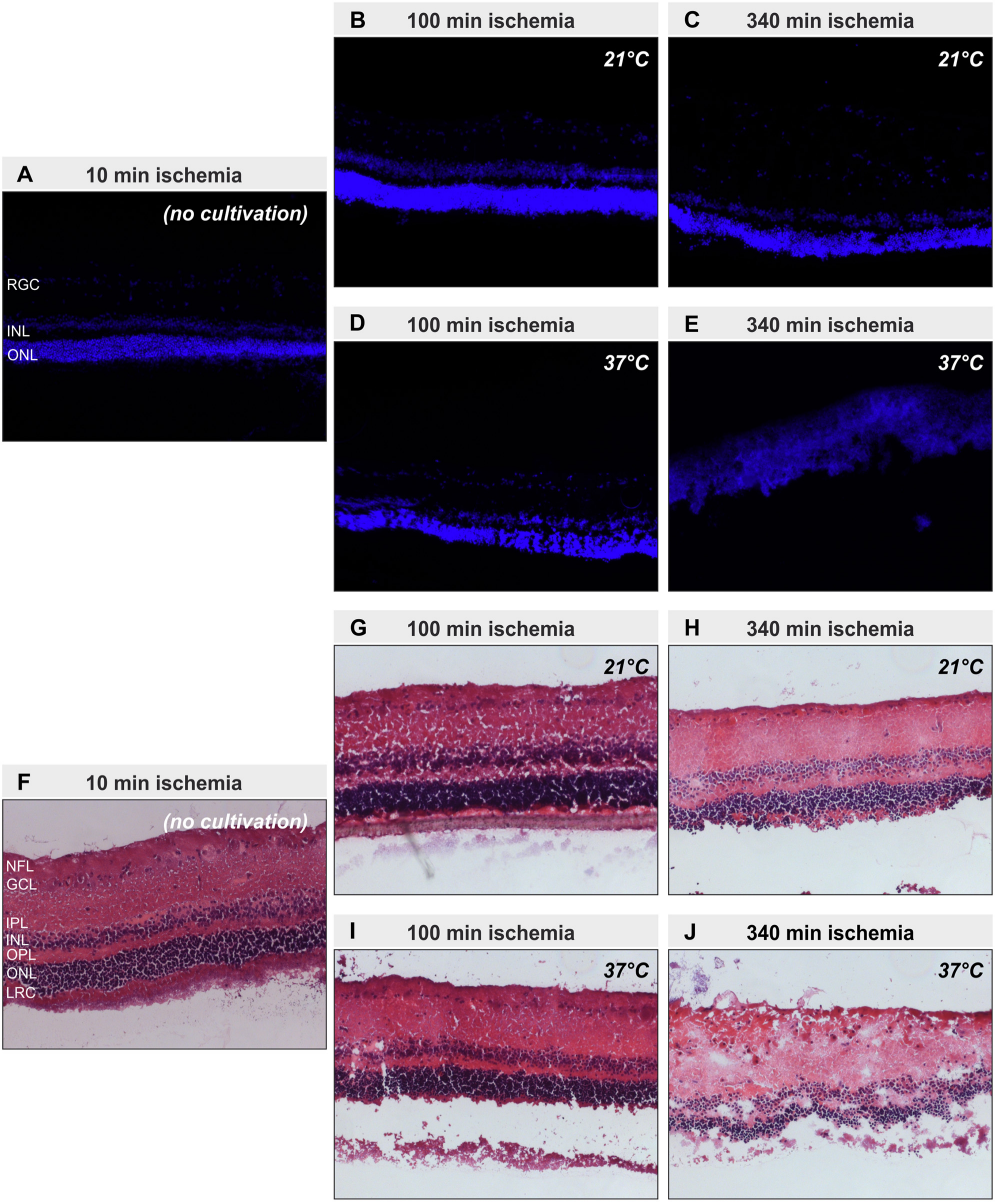
Retinal structure is preserved at 21°C storage. Representative pictures of cryosections of the five different groups (10 min ischemia (no cultivation) (A, F); 100 min ischemia at 21°C (B, G); 340 min ischemia at 21°C (C, H); 100 min ischemia at 37°C (D, I); 340 min ischemia at 340°C (E, J)) after staining with DAPI (A-E) or haematoxylin and eosin (F-J) to visualize nuclei and retinal structure are shown. Except for the probes with 340 min of ischemia stored at 37°C, which show a degenerated retina, all other sections show a preserved retinal structure. Layers of the retina: nerve fiber layer (NFL), ganglion cell layer (GCL), inner plexiform layer (IPL), inner nuclear layer (INL), outer plexiform layer (OPL), outer nuclear layer (ONL), layer of rods and cones (LRC)

Representative pictures of cryosections revealed preservation of the retinal structure in all eyes except for those stored at 37°C for 340 min (Fig 3). In these eyes the retinal architecture was severely disturbed (Fig 3E and 3J). The inner and outer nuclear layer could be hardly distinguished from each other, because the outer plexiform layer had nearly disappeared. Furthermore, the inner plexiform layer was thickened and some of the cells of the retinal ganglion cell layer were “dislocated” into the nerve fiber layer (Fig 3J). The severely disturbed retinal architecture goes in line with the observation during tissue preparation, that only in this group the retina was like a viscous fluid which could not be grasped anymore by forceps. All other groups still had the typical consistency of a retina.

## Discussion

The results of the presented study suggest that hypothermia can prolong the tolerance time of retinal ganglion cells against ischemia.

The analysis of the spontaneous retinal ganglion cell spikes (action potentials) showed that under hypothermic conditions the retinal ganglion cells can survive an acute ischemic insult of 340 min. With real-time PCR we could detect 5.8 fold more living retinal ganglion cells in the tissue when the eyes exposed to 340 min of ischemia were kept under hypothermic conditions during the ischemic period—even after subsequent 24h of cultivation. This conclusion can be drawn, because Thy-1 mRNA is exclusively produced by the retinal ganglion cells in the retina and the amount of Thy-1 mRNA expression can be used as equivalent to the number of living retinal ganglion cells[17]. In contrast to ischemia at body temperature hypothermia preserved the retinal architecture for many h as observable in the stained retinal cryosections (Fig 3C). Additionally in the group with 340 min of ischemia at 37°C the retina was like a thick, sticky fluid and no tangible tissue anymore. The results of the real-time PCR and the measurements of the spontaneous retinal ganglion cell responses are supported by the histological findings.

Interestingly the results of the RGC recordings (analyzed directly after preparation) and the Thy-1 mRNA (analyzed after 24 h of cultivation) differed especially in the 100 min ischemia at 37°C-group. This group showed a nearly fully depressed RGC-activity but a substantially retained expression of Thy-1 mRNA. The reason for this could be that the ischemic insult at the beginning leaves RGCs structurally intact but functionally inactive. The same effect occurs with neurons in the penumbra of an ischemic stroke[18,19]. This could explain, why the neurons were electrophysiological silent directly after the ischemic insult, but still somehow alive, because the Thy-1 mRNA expression was maintained even after 24h of cultivation.

By extending the retinal ganglion cell tolerance time to ischemia, hypothermia could open a therapeutic window for a causal therapy of acute retinal ischemic diseases such as central retinal artery occlusion. Nevertheless, hypothermia cannot ameliorate the impact of ischemia completely. The deleterious effect of ischemia is only slowed down and not stopped by hypothermia.

Already other studies showed that hypothermia is neuroprotective against ischemia [8,9,10]. Nevertheless, we are the first who analyzed the neuroprotective effect of hypothermia at the level of retinal ganglion cells. These retinal ganglion cells are the decisive cells for the visual outcome after an ischemic retinal insult in the clinical setting and therefore should be –in our opinion- the center of investigations.

Recently Salido et al. reported that global or local hypothermic preconditioning (32 and 33°C for 20 min) protects the rat retina from ischemic damage[20] on the level of retinal ganglion cells. However, preconditioning with hypothermia is difficult to achieve in real life as nobody can know in advance when an acute ischemic event will take place. Thus, inducing hypothermia as soon as possible after the onset of acute ischemia, by a cooled irrigation solution during vitrectomy for example, seems to be more promising. Vitrectomy can be performed within 15 min after diagnosis in local anesthesia.

Importantly hypothermia not only reduces the metabolism and energy consumption, but also protects cells against glutamate excitotoxicity, which is a nearly ubiquitous existing damaging mechanism in most acute and chronic ischemic retinal diseases[21,22]. Additionally hypothermia reduces the activity of neuronal-damaging neuroinflammatory cascades[23].

Our study was carried out on enucleated bovine eyes. Therefore, our results have to be confirmed in an in vivo model, before conclusions can be drawn for a clinical setting. Nevertheless in our model the retinal survival time could be extended to 340 min of ischemia when the retina was stored at 21°C during the ischemic period.

In conclusion, hypothermia protects retinal ganglion cells against an ischemic insult and prolongs their ischemic tolerance time. Hypothermia therefore could open a therapeutic window to initiate causal treatments of acute ischemic retinal diseases such as central retinal artery occlusion. Nevertheless it still has to be investigated which temperature of the irrigation solution results in the best neuroprotective effect.
